# Alumni Association of the American Veterinary College

**Published:** 1891-07

**Authors:** 


### ALUMNI ASSOCIATION OF THE AMERICAN VETERINARY COLLEGE.

### Officers for 1891—1892.

*President*, W. Horace Hoskins, D.V.S., 12 S. 37th Street, Phila., Pa.
*Vice-President*, Wm. H. Lowe, D.V.S., 188 Ellison Stree,t Paterson, N. J.
*Secretary*, E. B. Ackerman, D.V.S., 141 West 54th Street, New York.
*Treasurer*, Andrew Strange, D.V.S., 322 West 15th Street, New York.
*Librarian*, Th. Birdsall, D.V.S., 159 Crosby Street, New York.

### Executive Committee.

Dr. Theo. Birdsall, (*Chairman.*)
Drs. S. K. Johnson, L. H. Howard, A. G. Vogt, W. B. E. Miller, M. W. Drake, W. L. Labaw.

### Resident State Secretaries.

A. A. Tuttle, D.V.S., West Haven, Conn.
H. F. Lane, D.V.S., Havana, Cuba.
W. B. Rowland, D.V.S., Pasadena, Cal.
A. Edward Perry, D.V.S., (Colorado) Riverhead, L. I.
H. B. McDowell, D.V.S., Middleton, Del.
Alex. McKenzie, D.V.S., Washington, D. C.
G. S. Agersbog, D.V.S., Vermilion, Dak.
E. A. Child, D.V.S., Leesburg, Fla.
W. J. Elliott, D.V.S., Peoria, Ill.
Clement V. Elliott, D.V.S., Terra Haute, Ind.
A. B. Morse, D.V.S., Des Moines, Iowa.
Hara Toka Yokura, D.V.S., Tokio, Japan.
J. L. Kidd, D.V.S., Lexington, Ky.
D. C. Ayre, D.V.S., Leavenworth, Kan.
Austin Peters, D.V.S., Boston, Mass.
D. R. Hoffman, D.V.S., Baltimore, Md.
F. W. Huntington, D.V.S., Woodford, Me.
John T. See, D.V.S., Minneapolis, Minn.
T. E. White, D.V.S., Sedalia, Mo.
G. H. Keefer, M.D., D.V.S., Hilsdale, Mich.
R. F. Burleigh, Ph.G., D.V.S., Franklin, N. H.
J. F. Autereith, D.T.S., Jersey City, N. J.
Jos. Ogle, Jr., D.V.S., Greenpoint, (L. I.) N. Y.
Frank Harvey, D.V.S., Durham, N. C.

J. D. Fair, D.V.S., Berlin, Ohio.
Chas. T. Goentner, D.V.S., Bryn Mawr, Pa.
W. L. Burt, D.V.S., Providence, R. I.
J. W. Sheibler, D.V.S., Memphis, Tenn.
S. L. Richards, D.V.S., Salt Lake, Utah.
L. C. Wakefield, D.V.S., Montpelier, Vt.
J. A. Myers, D.V.S., Zinville, Va.
R. A. Stoute, D.V.S., Barbadois, W. T.
Salomon Bock, D.V.S., Cheyenne, Wyo.
J. B. Ford, D.V.S., Moorefield, W. Va.
Christmas Evans, D.V.S., Racine, Wis.
Louis J. Touissant, D.V.S., Milwaukee, Wis.

